# Soluble Isoform of Suppression of Tumorigenicity 2 (ST2) Biomarker in a Large Cohort of Healthy Pediatric Population: Determination of Reference Intervals

**DOI:** 10.3390/jcm11164693

**Published:** 2022-08-11

**Authors:** Marco Alfonso Perrone, Julien Favresse, Annamaria D’Alessandro, Federica Albanese, Coralie De Bruyne, Stefano Ceccarelli, Fabrizio Drago, Paolo Guccione, Ottavia Porzio, Benedetta Leonardi

**Affiliations:** 1 Department of Pediatric Cardiology and Cardiac Surgery, Bambino Gesù Children’s Hospital IRCCS, 00146 Rome, Italy; 2 Department of Cardiology and Cardio Lab, University of Rome Tor Vergata, 00133 Rome, Italy; 3 Department of Laboratory Medicine, Clinique Saint-Luc Bouge, 5004 Namur, Belgium; 4 Department of Pharmacy, Namur Research Institute for LIfes Sciences, University of Namur, 5000 Namur, Belgium; 5 Clinical Laboratory Unit, Bambino Gesù Children’s Hospital IRCCS, 00146 Rome, Italy; 6 Department of Pediatric Cardiology, Cliniques Universitaires Saint-Luc, 1200 Brussels, Belgium; 7 Department of Experimental Medicine, University of Rome Tor Vergata, 00133 Rome, Italy

**Keywords:** ST2, heart failure, pediatric population, reference intervals

## Abstract

**Introduction:** Only little data exists on ST2 reference intervals in healthy pediatric populations despite the high importance of this biomarker in adults with heart failure. The aim of the study was to assess the reference intervals of ST2 in a wide healthy pediatric cohort. **Methods:** We evaluated the serum concentrations of ST2 biomarker in 415 healthy pediatric subjects referred to our analysis laboratory. Subjects were categorized according to age (i.e., 0–6 (*n* = 79), 7–11 (*n* = 142) and 12–18 years (*n* = 191)) and sex. They were not suffering from any cardiac disorders, metabolic disorders, lung diseases, autoimmune disorders or malignancies. A written consent was obtained for each individual. No duplicate patients were included in the analysis and the presence of outliers was investigated. Reference intervals (Mean and central 95% confidence intervals) were determined. **Results:** Three outliers have been identified and removed from the analysis (60.0, 64.0 and 150.2 ng/mL). A total of 412 subjects were therefore included. The mean value for the whole population was 15.8 ng/mL (2.4–36.4 ng/mL). Males present a significantly higher mean concentration compared to females (17.2 versus 14.4 ng/mL, *p* = 0.001). A significant trend toward higher ST2 values with age was also observed, but for males only (r = 0.43, *p* < 0.0001). If considering age partitions, only males of 12–18 years (mean = 21.7 ng/mL) had significantly higher ST2 values compared to the other groups (ranging from 11.9 for males 0–6 years to 15.2 for females 12–18 years; *p* < 0.0001). **Conclusions:** We described age and sex-specific reference intervals for ST2 in a large healthy pediatric population. We found that ST2 values differ between sexes if considering all participants. A significant increase in ST2 with age was also observed, but only for males of 12–18 years.

## 1. Introduction

Heart failure (HF) represents a complex syndrome characterized by the reduction of the left ventricular function and by the alteration of neurohormonal regulation, which involves both adults and younger subjects. Along with the clinical symptoms of HF, risk factors and abnormal electrocardiogram, the diagnostic algorithm of HF also relies on the measurement of the type B natriuretic peptides (BNP or NT-proBNP). Its importance in the early diagnosis, risk stratification and follow-up of patients is well known [1]. However, even if the natriuretic peptides are able to identify, in an extremely sensitive way, the presence of hemodynamic overload and neurohormonal regulation caused by HF, they are produced and released as a reaction to generic damage. Hence, their variation may not correctly recognize the nature of the cardiac damage and identify in each patient the main cause of the disease progression. Other markers have been recently proposed in the clinical management of patients with HF, among which the most promising seems to be the soluble isoform of suppression of tumorigenicity 2 (ST2), belonging to the family of interleukin receptors [2,3,4]. Compared to other cardiac biomarkers (i.e., troponins and natriuretic peptides), its concentration is less influenced by kidney function and other conditions [5]. Baseline ST2 levels are strong predictors in HF at chronic and acute stages, independently from BNP levels. In adults, this biomarker is an independent risk factor for adverse events in patients with dilated cardiomyopathy and acute HF [6,7,8] and an independent predictor in clinically stable patients [9]. Furthermore, it also seems to play an important role in the prediction of all-cause mortality in high-risk adult patients with complex congenital heart disease (CHD) [10]. Even Geenen et al. have documented in a wide cohort of adult patients with CHD its significant association to adverse cardiovascular events [11]. The reference intervals for this biomarker seem to be different depending on the selected population, with higher values in healthy American individuals [12] than in Europeans [5]. Some studies highlighted that ST2 values are unaffected by age and are considerably higher in males compared to females [5,12]. Despite a different normal cut-off value found in the above-mentioned studies, depending on the nationality of the population, a ST2 number of 35 ng/mL seems to differentiate the adult patients with high risk from those with a lower one [13]. Even if the ST2 has been included as an additive risk stratification biomarker for acute and chronic HF in adults [14], there are only a few studies in pediatric populations on its possible use in heart disease [15,16] and on the definition of reference intervals for this population [17,18]. In addition, the existing studies on reference intervals only include a low number of pediatric participants. In this study, we aimed to establish reference intervals of ST2 in a wide healthy pediatric population, given that some studies suggest the important role of this biomarker in cases of HF among young pediatric patients.

## 2. Material and Methods

### 2.1. Study Subjects

We have prospectively selected the blood sampling of healthy pediatric subjects (<18 years old) from the Department of Laboratory Medicine of Bambino Gesù Children’s Hospital IRCCS from July to December 2019. Participants with known cardiac disorders, hypertension, metabolic disorders, respiratory diseases, autoimmune disorders or malignancies were excluded from the study. No duplicate patients were included in the analysis. Blood samples were taken from pediatric subjects who were referred for routine blood tests by pediatricians after a medical examination. Extra samples were obtained for study purposes. The study was approved by the Ethics Committee of the Bambino Gesù Children’s Hospital IRCCS (protocol code 1772/2019) and informed consent was obtained from the parents of each child. The study was conducted in accordance with the Declaration of Helsinki.

### 2.2. Biochemical Measurements

The blood samples were stored frozen at –80 °C before being analyzed. ST2 was measured using a sandwich ELISA kit (Presage^©^ ST2 assay, Critical Diagnostics, San Diego, CA, USA). A human ST2 standard calibrator was provided for this assay. ST2 concentrations were measured according to ST2 assay procedures described in the manual. Briefly, standard (100  μL) and diluted samples (1: 20 in sample diluent) were added to the well of a ready-to-use microtiter plate coated with mouse monoclonal anti-human ST2 antibody (60 min at room temperature). The standard curve was in the concentration range 3.1–200  ng/mL. Then, each well of the plate was incubated with biotinylated antibody reagent (100 μL, 60 min at room temperature by mixing at 750 rotations per minute (rpm) and with streptavidin-HRP conjugated (100  μL, 30  min at room temperature, by mixing at 750 rpm). Finally, we added the TMB substrate (20  min at room temperature in the dark, by mixing at 750 rpm) and stopped the reaction to read the absorbance at 450  nm. The ST2 ELISA immunoassay presents a limit of detection of 1.8 ng/mL and a limit of quantification of 2.4 ng/mL. Results below the LOQ were rounded to the LOQ.

### 2.3. Statistical Analysis

Descriptive statistics were used to analyze the data: count and percentage for categorical data and mean standard deviation, 95% CI of the mean for continuous data. Dixon-Reed and Tukey tests were used to detect potential outliers. Outliers were removed from the analysis. Reference intervals were calculated using the nonparametric rank method in case of partitions ≥ 120 sample size or using the robust method in case of partitions < 120 sample size [19], based on CLSI and IFCC C28-A3 guidelines; 90% CI around thr lower and upper limits were also calculated. Simple linear regression between ST2 concentrations and age according to sex were performed. Pearson’s correlation coefficients were used to investigate the strength of the relationship between ST2 and age. The Gaussian distribution of data was also verified. The difference between females and males for ST2 (without age partitions) was performed using an unpaired *t*-test. The difference between age categories was assessed using an ordinary ANOVA with Tukey multiple comparison tests. Statistical analyses were performed by using STATA software (version 14.1, College Station, TX, USA) and GraphPad Prism software (version 9.3.0, San Diego, CA, USA). All tests were two-sided and *p* < 0.05 was used as a significance level.

## 3. Results

**Figure 1:**
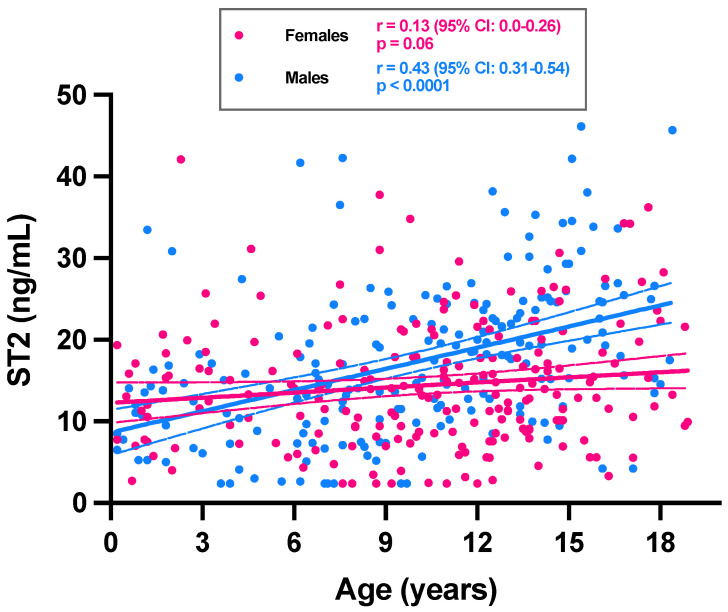
ST2 concentrations among 412 pediatric subjects as a function of age and sex. Trend for ST2 as a function of age is expressed for females (pink dots) and males (blue dots). Simple linear regressions are represented with continuous lines. Dotted lines correspond to the 95% CI of the linear regression.

Three outliers have been identified and removed from the analysis: one female of 16 years (i.e., 150.2 ng/mL), one male of 2 years (i.e., 64.0 ng/mL) and one male of 15 years (60.0 ng/mL). A total of 412 subjects were enrolled for the study, of which 212 were females (51.5%). Sera from three age ranges were collected: 0–6 years (79 patients; 41 females), >6–11 years (142 patients; 68 females) and >11–18 years (191 patients; 103 females). Table 1 displays mean ST2 results and its 95% confidence interval per age range and sex. The mean ST2 concentration for the entire group was 15.8 ng/mL (and a median of 14.7 ng/mL). Means in females and males were significantly different (14.4 versus 17.2 ng/mL; *p* = 0.001) (Table 1). A total of 14 subjects had ST2 concentration below the LOQ (i.e., 2.4 ng/mL). Six were females (2.8%) and 8 were males (4.0%). All subjects with ST2 < 2.4 ng/mL were less than 12 years of age (Figure 1).

**Figure 2:**
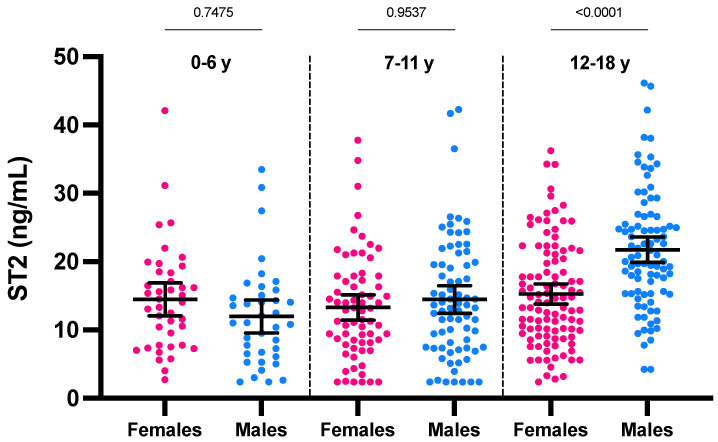
ST2 concentrations according to age partitions and sex. Mean and 95% CI of the mean are represented in black.

If considering the ST2 values according to age, a positive and significant trend toward higher ST2 concentrations with age was only observed for males (r = 0.43; *p* < 0.0001) (Figure 1). The analysis stratified according to age groups showed a statistically significant difference in ST2 means between sexes only in category 12–18 years (21.7 ng/mL for males versus 15.2 ng/mL for females; *p* < 0.0001) (Figure 2). The male group of 12–18 years was also significantly different from all other groups (*p* < 0.0001). In females of less than 18 years, we therefore found no rationale for using intermediate age reference intervals. Nevertheless, the application of adapted reference intervals for males of 12–18 years seems appropriate in comparison to younger males (i.e., <12 years).

## 4. Discussion

The determination of reference intervals is paramount in the assessment of patient health and in clinical decision making [19,20]. Compared to the reference intervals in adults that are well established, reference intervals in pediatric populations are mostly incomplete. The use of reference intervals derived from adults can be inappropriate and can lead to misdiagnosis and/or to inappropriate treatment. There is therefore a need for developing reference intervals that are specific to the pediatric population [19]. Additionally, reference intervals should ideally be studied according to age and sex.

In the present study, we aimed at evaluating the reference intervals of the ST2 biomarker in a healthy pediatric population. For that purpose, a total of 412 donors (212 females and 200 males) were included with a large range of ages, spanning from 1 month to 18 years. Another strength of our study is that the design to obtain reference intervals was based on CLSI and IFCC C28-A3 guidelines [21]. As for other biomarkers in pediatrics, only a few studies have determined the reference intervals for ST2, with a lower number of included subjects compared to our study.

Several studies have shown the interest of measuring ST2 in a pediatric population. Emerging data support the use of ST2 for diagnosis, monitoring and prognostication of pediatric heart disease [22,23]. Additionally, its concentration has been shown to be related to the severity and worsening of pulmonary arterial hypertension [24]. ST2 is also a predictor of readmission after congenital heart surgery [25,26] and it can assess the risk factor of graft-versus-host disease [27]. Further studies are however needed to confirm the interest of measuring ST2 in some specific pediatric populations, alone or in combination with other cardiac biomarkers (i.e., troponins, natriuretic peptides, galectin-3).

Using the same ST2 assay (sandwich ELISA kit from Presage^©^ ST2 assay, Critical Diagnostics, San Diego, CA, USA), the median ST2 value obtained in our pediatric cohort (14.7 ng/mL) was lower than the one reported by Meeusen et al. (i.e., 21 ng/mL) [18], Caselli et al. (i.e., from 16.6 to 23.0 ng/mL) [17] and Hauser et al. (i.e., 17.7 ng/mL) [15]. This could be explained by the number of samples analyzed, the absence of outlier elimination, the different age ranges considered and by the different populations enrolled. In fact, the three above-mentioned studies enrolled smaller cohorts compared to our study (from 89 to 240 participants) [15,17,18]. Sex differences in ST2 levels were observed in our study with higher values in males. This evidence is in disagreement with the study of Caselli et al. which showed a ST2 independence from age and gender in a smaller population [17]. On the contrary, Meeusen et al. also found a significant positive correlation with ST2 values and age only among males, with higher ST2 values in males compared to females only from 15 years of age [18]. The absence of correlation between ST2 and age that Caselli et al. found can also be explained by a higher proportion of younger subjects (75% were <12 years) [17]. Interestingly, the population enrolled by Meeusen et al. had a higher percentage of older participants [18]. In our study, the higher proportion of pediatric subjects was >12 years (46.4%). Studies performed in both healthy adults and in HF patients also support our results about higher levels of ST2 in males compared to females [5,12,28]. Given that various sex-specific hormones, such as testosterone and estradiol, could be involved in modulating plasma concentration of ST2, its rise in males may become evident from puberty [29]. The link between ST2 and the expression of various hormones deserves further investigations. Interestingly, using a cohort of 94 pediatric patients with dilated cardiomyopathy, You et al. also observed a gradual increase in ST2 levels with age [16]. Of note, only 14 subjects out of 412 had ST2 results below the LOQ of the assay. These latter individuals were all under 12 years.

## 5. Conclusions

In conclusion, and to the best of our knowledge, our investigation is the largest prospective study to determine reference intervals in a cohort of pediatric participants for ST2. Our results highlighted that ST2 values are not age-dependent in females, while in males, ST2 levels tend to increase in subjects aged 12–18 years. Our results will allow improved study into the utilization of ST2 biomarkers in pediatric patients with heart disease.

## Figures and Tables

**Table 1:** ST2 results in ng/mL for all participants and categorized per age and sex. Mean, standard deviation, 95% confidence intervals of the mean, reference intervals and 90% CI of lower and upper limits are represented.

	*n*	Median	Mean	SD	95% CI of the Mean	Reference Interval	90% CI for Lower Limit of RI	90% CI for Upper Limit of RI
All donors	412	14.7	15.8	8.7	14.9–16.6	2.4–36.4	2.4–2.7	34.3–40.5
All females	212	13.3	14.4	7.7	13.3–15.5	2.4–34.3	2.4–3.2	29.6–37.8
All males	200	15.7	17.2	9.5	15.9–18.5	2.4–41.6	2.4–2.7	35.3–45.7
Females 0–6 y	41	14.1	14.5	7.7	12.0–16.9	3.4–33.6	2.4–4.9	28.0–39.7
Males 0–6 y	38	11.0	11.9	7.5	9.5–14.4	1.8–31.7	1.0–3.3	26.0–38.7
Females 7–11 y	68	12.9	13.2	7.8	11.3–15.1	2.4–35.6	2.4–2.5	25.6–37.8
Males 7–11 y	74	13.4	14.4	8.8	12.4–16.5	1.8–34.2	2.4–2.6	29.8–38.4
Females 12–18 y	103	14.1	15.2	7.7	13.8–16.7	3.4–33.8	2.6–4.5	30.5–37.0
Males 12–18 y	88	20.8	21.7	8.8	19.9–23.6	6.3–40.6	4.6–8.1	37.2–44.3

## Data Availability

The data presented in this study are available on request from the corresponding author, subject to authorization by the Scientific Direction of the Bambino Gesù Children’s Hospital IRCCS.
